# Hemispheric transfer and dyslexia: testing the deficit hypothesis for word and symmetry recognition using visual half-field tasks

**DOI:** 10.1186/s11689-026-09674-4

**Published:** 2026-01-29

**Authors:** Zita Meijer, Emma M. Karlsson, Robin Gerrits, Guy Vingerhoets, Helena Verhelst

**Affiliations:** 1 Department of Experimental Clinical and Health Psychology, Ghent University, Henri Dunantlaan 2, Ghent, 9000 Belgium; 2 Research Group Cognition and Plasticity, Max Planck Institute for Human Cognitive and Brain Sciences, Leipzig, Germany

**Keywords:** Developmental dyslexia, Interhemispheric transfer deficit, Visual half-field tasks, Lexical decision task, Symmetry decision task

## Abstract

**Background:**

The interhemispheric transfer deficit theory proposes that individuals with dyslexia have impaired interhemispheric transfer, particularly affecting the integration of visual information from the left and right visual fields. This study aimed to evaluate this hypothesis by examining interhemispheric transfer in dyslexia using visual half-field tasks targeting both linguistic and visuospatial processing.

**Methods:**

We examined interhemispheric transfer in dyslexia using two visual half-field tasks: a lexical decision task to assess written word processing, and a symmetry decision task to examine visuospatial processing. We compared reaction times and accuracy in 90 Dutch-speaking participants (45 with dyslexia, 45 controls) across left, right, and bilateral stimulus presentations.

**Results:**

While both tasks successfully captured expected visual half-field differences in the control group, favoring the right visual field in the lexical decision task and the left visual field in the symmetry detection task, we did not observe that the dyslexia group showed increased differences between the two fields, as predicted by the interhemispheric transfer deficit theory. Furthermore, the dyslexia group benefited just as much as controls from stimuli presented simultaneously to both visual fields. Thus, no evidence of interhemispheric transfer deficits related to dyslexia was found in either task.

**Conclusions:**

These findings challenge the broad applicability of the interhemispheric transfer deficit theory in dyslexia, suggesting that such impairments may be task-dependent rather than domain-general. Future studies should further explore the conditions under which interhemispheric transfer deficits might occur in dyslexia.

**Supplementary Information:**

The online version contains supplementary material available at 10.1186/s11689-026-09674-4.

## Introduction

Dyslexia, also referred to as developmental dyslexia, is a common neurodevelopmental learning disorder characterized by persistent reading and spelling difficulties, despite otherwise normal development [40]. These reading problems manifest as difficulties with identifying written words and with fluent reading [38] and deficits in phonological awareness [10]. Dyslexia is a multifaceted disorder that presents with varying symptom severity and often co-occurs with other learning disorders [43]. Multiple theories have been developed to understand its underlying etiology, but the exact causes remain unknown. There is, however, consensus that dyslexia entails a combination of neurobiological, genetic, and environmental factors [27].

One neurobiological theory that has gained some support is the interhemispheric transfer deficit theory, which proposes that the reading difficulties observed in individuals with dyslexia may be attributed to an impaired interhemispheric information transfer between the two hemispheres through the corpus callosum [2, 46]. This stems from the assumption that written-word processing is predominantly performed by inferior frontal and posterior temporal regions in the left hemisphere, and that visual information arriving in both hemispheres must be integrated in these areas to facilitate reading [26]. A defect in the corpus callosum would disrupt this process by impairing the efficient transfer of information between the brain’s two hemispheres [25]. This disruption would not only affect reading-related processes, but would also impact visuospatial processing, which instead, is processed in the right hemisphere and also requires integration of visual information from both hemispheres [2, 9, 64]. Supporting the role of interhemispheric transfer in visuospatial processing, Badzakova-Trajkov et al. [2] examined children with dyslexia using a simple reaction time task to visual stimuli presented to the left, right, or both visual fields. They found that the dyslexia group showed enhanced redundancy gain when responding with the left hand, suggesting atypical corpus callosum function and asymmetrical hemispheric transfer. Although other studies report conflicting results on the relationship between dyslexia and impaired visuospatial ability, a meta-analysis suggests that dyslexic groups typically score worse on visuospatial tasks [13].

Neuroanatomical studies showing structural abnormalities in the posterior parts of the corpus callosum in individuals with dyslexia [21, 55] suggest some validity for the interhemispheric transfer deficit theory. However, no study has directly linked structural differences in the corpus callosum to impairments in interhemispheric transfer in dyslexia. Some behavioral studies have examined interhemispheric transfer in dyslexia, but reported inconsistent findings for both language (in adults: [7, 30], and in children: [8]) and visuospatial tasks (in adults: [63], and in children: [18, 22]). For example, Henderson et al. [30] and Bradshaw et al. [7] both used the visual half-field technique to examine interhemispheric transfer in adults with dyslexia. Because information from the right visual field (RVF) projects to the left visual cortex and information from the left visual field (LVF) projects to the right visual cortex, words perceived in the LVF must be transferred to the left hemisphere, where word-level processing primarily occurs [26]. This transfer, which occurs via the corpus callosum, is theorized to cause a delay [59] that can be observed in behavioral data [29, 33]. Consequently, the visual half-field (VHF) method can serve as a behavioral indicator of which hemisphere primarily processes different categories of visual information, as well as a tool to examine transfer time between the two hemispheres. For example, when written words are briefly presented in the RVF, participants typically require less time to report them compared to when words are presented in the LVF [45]. This difference is due to the dominant role of the left hemisphere in language processing, a phenomenon known as the right visual field advantage in word processing [4, 6, 29]. On the other hand, visual half-field tasks typically show a left visual field advantage in visuospatial tasks, such as symmetry detection, due to the right hemisphere’s dominance in visuospatial processing [33, 64].

In addition, presenting stimuli bilaterally can further improve performance in VHF tasks, a phenomenon known as a ‘redundant bilateral advantage’ (RBA; [29]). Seeing a word in both visual fields can result in faster reaction times compared to when it is presented in only one field [41]. Although the preferred visual field typically allows quicker processing in unilateral presentations, presenting stimuli in both fields is thought to accelerate processing even further by incorporating information from the non-preferred field [41]. While not all studies support this effect [19, 36], the absence of a RBA in split-brain patients, whose commissural pathways are surgically severed, is at least suggestive that interhemispheric communication from the non-preferred field is key for this advantage [42].

To examine the interhemispheric transfer deficit theory in dyslexia, Henderson et al. [30] and Bradshaw et al. [7] used VHF tasks in which adults (aged 18–46) were asked to perform a word reproduction task, by typing words presented to the LVF, the RVF, or bilaterally (shown in both fields). Both studies found that individuals with dyslexia performed significantly worse when words were presented in the LVF compared to non-reading-impaired controls. This LVF-specific difference in processing time also resulted in a greater disparity in accuracy scores between the RVF and the LVF. This finding aligns with the interhemispheric transfer deficit theory, suggesting that impaired processing of words in the LVF results from difficulties in transferring visual information from the right hemisphere. In addition, Henderson et al. [30] did not find an RBA in word processing in their dyslexia group, attributing this to delayed transfer from the LVF (right hemisphere) to the left hemisphere, which prevented right-hemisphere facilitation. However, Bradshaw et al. [7] found that the dyslexia group had a RBA that was comparable to the control group, failing to replicate the lack of an RBA in dyslexia. Nevertheless, Bradshaw et al.’s [7] findings remain consistent with the interhemispheric transfer deficit theory, as the authors suggest that the poorly transferred information from the LVF may still facilitate processing in the left hemisphere to some extent.

While both Henderson et al. [30] and Bradshaw et al. [7] provide behavioral evidence supporting the interhemispheric transfer deficit theory in dyslexia, several key aspects remain unexplored in the literature. First, the authors measured only accuracy, as their typing task did not assess reaction time. However, since the interhemispheric transfer deficit theory concerns processing speed, reaction times would provide a more informative measure. Second, by focusing exclusively on verbal tasks, both studies were unable to determine whether interhemispheric transfer deficits in dyslexia extend beyond the verbal domain to visuospatial processing, as suggested by Badzakova-Trajkov et al. [2]. Addressing these key aspects would improve our understanding of the validity and scope of the interhemispheric transfer deficit theory in dyslexia. Thus, the current study aimed to extend the findings of Henderson et al. [30] and Bradshaw et al. [7]. First, by using an alternative lexical decision VHF task, which allowed for the measurement of both accuracy and reaction times, to study the interhemispheric transfer in relation to processing speed. Second, by incorporating a second, non-verbal symmetry decision VHF task to examine whether interhemispheric transfer deficits in dyslexia extend to visuospatial processing.

Various tasks for studying lexical and symmetry processing are used in the literature. Lexical processing can be examined through word naming, same–different word matching, or the more widely-used word–nonword lexical decision task [3, 14, 15]. Lexical decision tasks can also differ in whether words are compared to pronounceable pseudowords or unpronounceable nonwords [28]. Because these tasks require written word recognition and individuals with dyslexia experience difficulties with processing written words [38], lexical decision tasks are particularly suitable for studying group differences between individuals with dyslexia and control participants [1, 39]. Likewise, symmetry perception tasks vary in the literature, including vertical, horizontal, or diagonal symmetry decisions and same-different judgments [65, 66]. These tasks require visuospatial processing, which is sometimes also considered to be impaired in individuals with dyslexia [13]. In the current study, we used a word–nonword lexical decision task and a vertical symmetry decision task, as these tasks are well-established in the visual half-field literature for capturing lateral asymmetries [31, 64].

The current study had three hypotheses. First (H1), we expected that the difference between the RVF and the LVF would be larger in the dyslexia group than in the control group for both VHF tasks, in both accuracy and reaction time, due to increased transfer time for stimuli presented to the non-dominant hemisphere. Secondly (H2), we predicted that the RBA would be reduced in the dyslexia group relative to controls for both accuracy and reaction times across both VHF tasks. Additionally (H3), we predicted that greater transfer delays would be related to dyslexia symptom severity, as measured by reading and spelling efficiency tests. Hypotheses 1 and 2 represent the primary predictions of the study, while hypothesis 3 is a secondary prediction.

## Method

### Participants

A total of 90 individuals took part in this study: 45 adults with a dyslexia diagnosis and 45 individuals without any diagnosis of language or reading impairment as controls. Participants were recruited through social media, word of mouth, and a student participation platform. Additional inclusion criteria were Dutch as a first language, age between 18 and 40 years, and right-handedness. All participants had normal or corrected-to-normal vision and no history of brain injury or neurological disease. It was confirmed that all individuals in the dyslexia group had an official diagnosis of dyslexia from a trained professional. For students, each completed grade was counted as one year of formal education. For the other adults, years of formal education were summed based on completed primary, secondary, and higher education. The two groups did not differ significantly in age, *t*(87.97) = 0.50, *p* = 0.620, nor in number of years of formal education, *t*(87.92) = −0.43, *p* = 0.665, and were perfectly matched for sex (see Table 1). The number of participants was based on the power analysis by Bradshaw et al. [7], who recommended a minimum of 30 participants per group, based on the data of Henderson et al. [30]. All participants were compensated; students received course credit for their participation, while all other participants received monetary compensation. The study was approved by the Medical Ethics Committee of Ghent University Hospital (approval number BC-09822).

**Table 1:** Participant demographics for the dyslexia and control groups

Demographics	Participant group	
	Dyslexia (*n* = 45)	Control (*n* = 45)
Mean age (SD)	22.31 (5.13)	21.78 (5.04)
Mean years in education (SD)	13.44 (1.91)	13.62 (1.97)
Females (%)	28 (62.2%)	28 (62.2%)

### Materials and procedures

#### Visual half-field tasks

Two VHF tasks were used in this study: a lexical decision task and a symmetry decision task. During the lexical decision task, participants had to decide whether a string of letters formed a word or not. During the symmetry decision task, they were instructed to decide whether each figure presented was symmetrical or not. Both tasks followed identical procedures (but used different stimulus types) and were designed according to the guidelines provided by Hunter and Brysbaert [31] for creating reliable behavioral VHF tasks. During the tasks, stimuli were presented to the left visual field (LVF), the right visual field (RVF), or to both visual fields simultaneously.

The stimuli of the lexical decision task consisted of 80 five-letter words and 80 five-letter non-words, selected from the Dutch Lexicon Project 2 [11] and the Dutch Lexicon Project [35], respectively. For the word-items, only nouns that were correctly identified on 100% of trials in a previous study [11] were selected to ensure high familiarity and minimize ambiguity. The non-words all had an accuracy rate ranging from 95 to 96% [35]. The stimuli were presented in the font ‘Fixedsys’, which ensured each word always had the identical length of 2.8 cm. The font size was set to 0.85 cm.

**Fig. 1:**
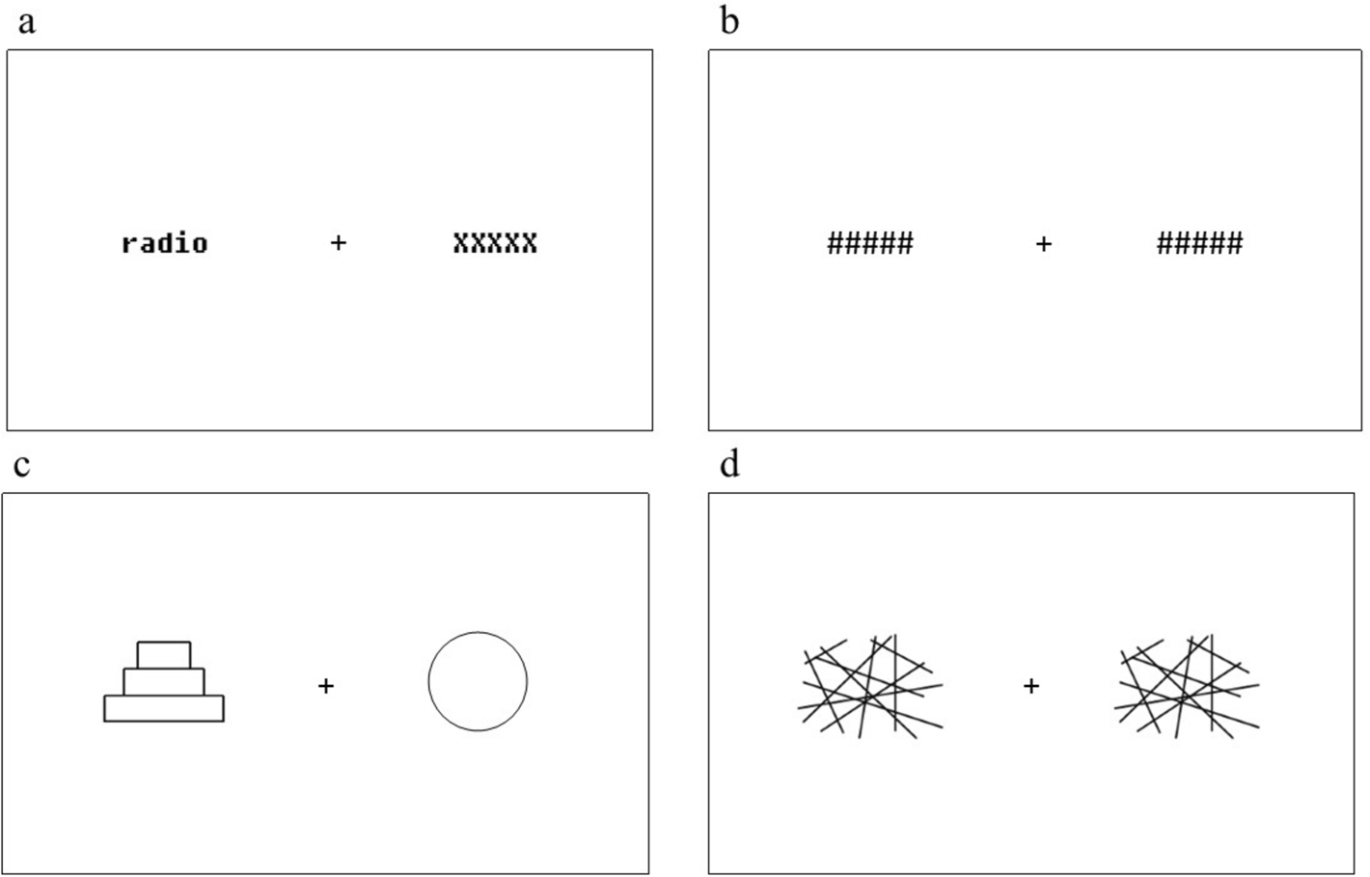
Examples of the stimuli and masks used in the two visual half-field tasks. Note: Examples of the stimulus dispay of **a** a LVF trial for the lexical decision task and **c** the symmetry decision task with stimuli on the left and placeholder on the right, and **b** the mask of the lexical decision task and **d** the mask of the symmetry decision task

For the symmetry decision task, the stimuli consisted of 60 symmetrical and 60 asymmetrical figures (see Fig. 1 for an example), designed based on the study by Verma et al. [64]. The stimuli had a width of 2–3 cm and a height of 1.5–2 cm. Some figures were repeated to achieve 80 trials with symmetrical and asymmetrical figures per condition.

For both tasks, the stimuli appeared 1.5 cm from the fixation cross to ensure that the stimuli were presented in the parafoveal visual field, defined as approximately 4 degrees of visual angle from the fixation point [56]. The stimuli were presented in black on a light grey background. The 160 stimuli of each task were each presented once in the LVF, once in the RVF, and once bilaterally, resulting in 480 trials per task. The trials were presented in a random order.

Both VHF tasks were run in Psychopy2 [48]. Participants were positioned 60 cm from a 15-inch laptop screen. At the start of each trial, a fixation cross was presented in the centre of the screen. A stimulus would appear after 500 ms and would be visible for 200 ms, after which a mask (‘#####’ for the lexical decision task and crosshatching lines of 2.5 cm × 2 cm for the symmetry decision task; see Fig. 1b and d) was presented for 200 ms to avoid an afterglow. For unilateral presentation trials, a placeholder was shown on the other side for a balanced visual presentation, in the form of ‘XXXXX’ for the lexical decision task and a circle with a diameter of 2 cm for the symmetry decision task (see Fig. 1a and c). The participants had to place their index and middle fingers of both hands on marked keys on a free-standing keyboard (‘J’ and ‘F’ for the index finger, and ‘E’ and ‘I’ for the middle finger). To respond, they were instructed to press both index fingers when they saw an existing word or a symmetric figure, or both middle fingers if they saw a non-word or a non-symmetric figure. Participants were instructed always to use both hands to answer, as response accuracy and reaction times can be influenced by the (in)congruence between stimulus location and responding hand [58]. An experimenter was present during the task to ensure participants used both hands to answer and to remind them of this rule if necessary. The experiment was self-paced, but participants were instructed to respond as quickly and accurately as possible. The subsequent trial started after a response was recorded. Participants were asked to focus on a fixation cross in the middle of the screen throughout the trial to maintain attention on the center of the screen and avoid eye movements towards the stimuli.

Both tasks began with 20 practice items, which were randomly selected from the 480 trials. After the practice trials, participants received feedback in the form of a score of 20. The experiment itself consisted of 4 blocks of 120 trials per task. Participants could take a self-paced break between blocks by pressing the spacebar to resume the task. All participants started with the lexical decision task, followed by the symmetry decision task. Participants completed both VHF tasks in about 30 min.

#### Reading and spelling tests

After the VHF tasks, a battery of reading and spelling tests was also administered to assess the participants’ language skills. The tests were selected based on Tops et al. [61], who identified Dutch word reading, Dutch word spelling, and phonological awareness as the most important predictors of dyslexia in higher education in Flanders. The administration of these tests took approximately 30 min.

##### Spelling

Spelling was assessed using a subtest of the advanced reading and spelling test, the GL&SCHR (Test voor Gevorderd Lezen & Schrijven [Test for Advanced Reading and Writing]; [20]). This subtest consists of 30 Dutch words that do not follow standard Dutch spelling rules, presented via an audio recording read at a rate of one word every 2 s. Participants were required to write down each word with the correct spelling. After the presentation, participants had the opportunity to complete any missed words and correct possible mistakes. Following this, participants indicated whether they were ‘unsure’, ‘almost sure’, or ‘very sure’ about the correctness of their spelling of each word. These confidence ratings were then combined with spelling accuracy to produce a weighted score for word spelling. The weighted score ranges from 0 to 150, with higher scores indicating better performance. According to Tops et al. [61], the weighted score has an effect size (*d*) of 2.28 in distinguishing between students with and without dyslexia, which is larger than the effect size of the number of correctly written words alone (*d* = 2.05).

##### Phonological awareness

Another subtest of the GL&SCHR [20] was used to measure phonological awareness. This subtest was a spoonerism task, in which participants had to switch the first phoneme of two spoken words (e.g., ‘Harry Potter’ becomes ‘Parry Hotter’). The words were presented via an audio recording, and participants responded orally with the two words containing the reversed first letters. The task consisted of six practice items and 20 test items. The total time taken to complete the test items was recorded, as recommended by Tops et al. [61], who suggest time over accuracy as the primary measure. The time score of the spoonerism task has an effect size (*d*) of 1.42 in distinguishing individuals with dyslexia from those without [61].

##### Reading

To assess word reading speed, the LEMs or Leestest 1-minuut studenten (Word Reading Test for students; [62]) was used. This test consists of a list of 132 Dutch words, and participants were instructed to correctly read aloud as many words as possible in 1 min. The words are arranged in increasing difficulty, as they become progressively less frequent. Scores for this test range from 0 (no words read correctly in one minute) to 132 (all words read correctly in one minute). This score has an effect size of *d* = 1.97 in distinguishing individuals with dyslexia from those without [61]. Inter-rater reliability for the reading test was ensured through a calibration session, during which experimenters agreed on the criteria for correct pronunciation.

### Analyses

All analyses were conducted in R (version 4.3.1; [51]), and figures were generated using the ggplot2 [67] and yarrr [49] packages. The accuracy scores and reaction times to words and symmetrical figures were analyzed, as this study focuses on word and symmetry detection and hemispheric processing differences related to these two specific categories. Including scores to non-words or non-symmetric stimuli could introduce additional variability related to different task demands and additional processing strategies, rather than reflecting the efficiency of interhemispheric transfer during the tasks [37]. Prior to statistical testing, outliers were removed based on individual participants’ reaction times. Trials with reaction times shorter than 250 ms were excluded, as these are considered too brief to reflect decision-related responses [5]. Additionally, trials with reaction times more than 2.5 standard deviations above or below a participant’s mean reaction time were excluded [52]. The average percentage of outliers removed was 0.03%. For further analyses, only reaction times from correct responses were included. Accuracy rates were converted into percentage scores.

For all statistical tests, an alpha level of 0.05 was used. Multiple comparisons were accounted for by Bonferroni-corrected *p*-values. Normality assumptions were visually checked using the *car* package in R [23]. To test the first hypothesis—that the difference between the two visual fields was larger in the dyslexia group —difference scores between the LVF and RVF were calculated for each participant, using both accuracy percentage scores and reaction time, respectively. As this was a directional hypothesis expecting a larger difference in the dyslexia group, one-tailed independent sample t-tests were used to compare the difference scores between the groups.

To test the second hypothesis, which predicts reduced RBA in the dyslexia group, an RBA score was first calculated for each participant. The presence of the RBA is reflected in higher accuracy for bilateral presentation than for presentation to the preferred visual half-field. The preferred visual field was determined using the difference scores described above. Based on the valence of the differences, it was determined whether participants showed a bias toward the RVF or the LVF (indicated by greater accuracy or reduced reaction times in that field). We used each individual’s preferred visual field to account for individual differences in hemispheric dominance. To calculate the RBA based on accuracy, the accuracy percentage scores for trials in the preferred visual field were subtracted from the accuracy percentage scores for bilaterally presented trials for each participant. The RBA of reaction time was calculated in the same way as for accuracy, but with reaction times for correct trials. Again, one-sided independent-samples t-tests were used to compare the calculated difference scores between the two groups, as we had a directional hypothesis predicting a reduced RBA in the dyslexia group. For all t-tests, Cohen’s *d* and the 95% confidence intervals of the estimated mean difference are reported.

To test the third hypothesis concerning the relationship between impaired interhemispheric transfer in individuals with dyslexia and their performance on reading and spelling tests, partial correlations were calculated between the language test scores and the difference in reaction times and accuracy between the RVF and the LVF in the dyslexia group. These analyses controlled for duration of education and sex, as duration of education is related to language abilities [16], and because women tend to score systematically higher on spelling and reading tests [54]. In addition, correlations between the RBA based on reaction times and accuracy, and the language test performance were tested in the dyslexia group, again controlling for education and sex. This was tested only within the dyslexia group because the hypothesis specifically concerned this group, and including the control group could have confounded the results due to differences in group-level performance.

## Results

### Language ability scores

Mean scores and standard deviations for the different language tests are displayed in Table 2. Participants in the control group showed superior performance on the test of spelling, *t*(87.73) = 9.48, *p* < 0.001, phonological awareness, *t*(58.26) = −5.78, *p* < 0.001, and the LEMs reading test, *t*(87.20) = 9.64, *p* < 0.001.

**Table 2:** Means, standard deviations, and effect size (Cohen’s d) of the language test scores

Language Test	Mean (SD)		Effect Size (*d*)
	Control	Dyslexia	
Spelling GL&SCHR	121.38 (12.23)	96.22 (12.93)	2.00^**^
Phonological Awareness (Spoonerisms)	103.73 (31.59)	164.49 (66.33)	−1.22^***^
LEMs (words/min)	105.69 (16.52)	73.63 (15.00)	2.03^***^

*SD* standard deviation, *GL&SCHR* Test voor Gevorderd Lezen & Schrijven [20], *LEMs* Leestest 1-minuut studenten [62], Spoonerisms: lower score equals a better performance

^**^ = *p* <.01, ^***^ = *p* <.001

### Visual half-field tasks

#### Difference between visual fields

**Fig. 2:**
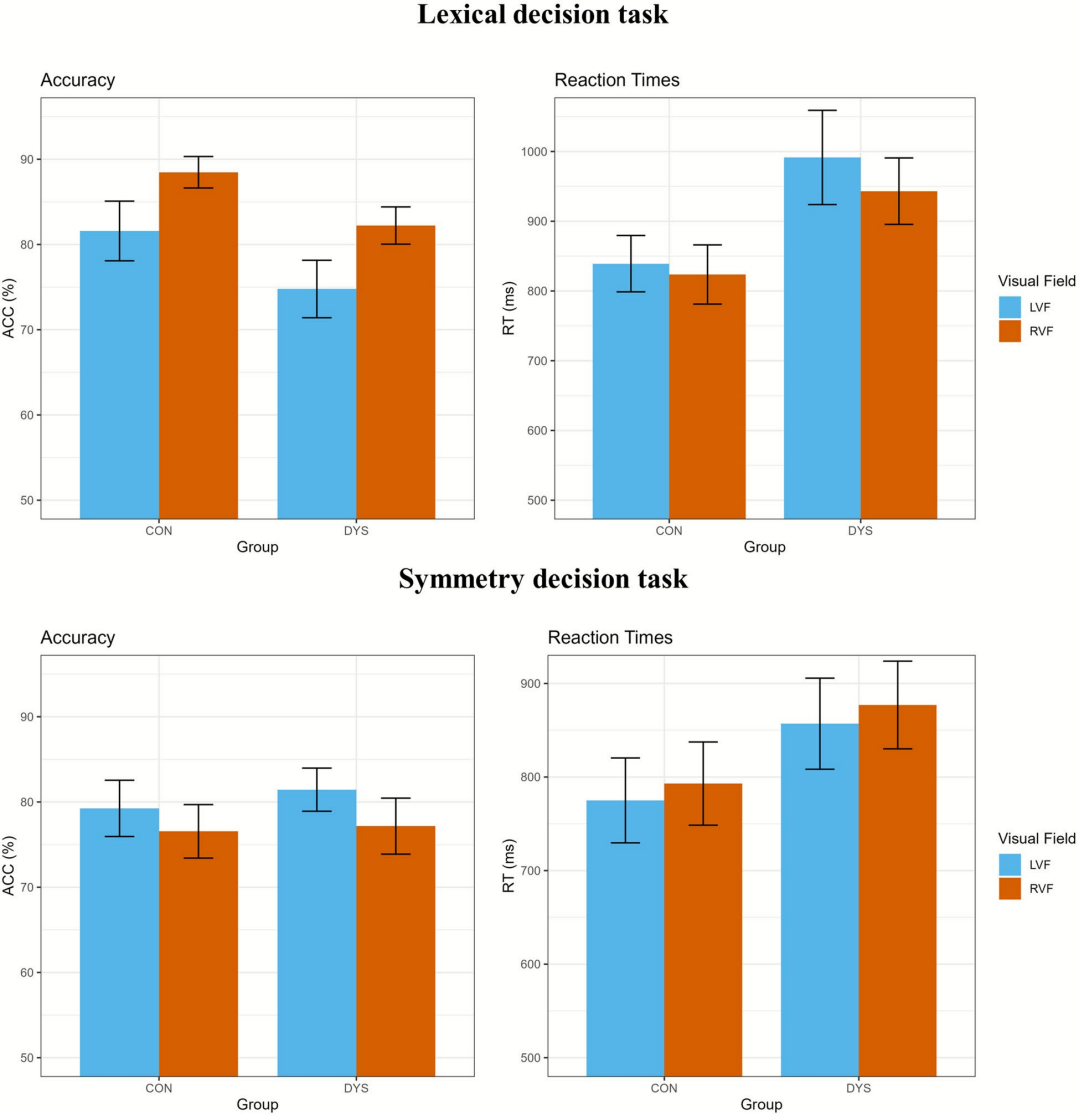
Bar plots of the accuracy and reaction times of left and right visual field of both tasks per group. Note: Bar plots of the mean accuracy (in %) and mean reaction times (in ms) per visual half-field condition per group, with 95% confidence intervals in black. Bars start at 50% accuracy and 500 ms reaction time. ACC = accuracy, RT = reaction time, RVF = right visual field, LVF = left visual field, CON = control group, DYS = dyslexia group

Mean accuracy percentage scores and reaction times for each visual half-field condition are summarized for each group and task in Table 3 and Fig. 2. As shown in Supplementary Figures S1 and S2, RVF–LVF difference scores varied within the dyslexia group, with some individuals showing larger differences and others smaller ones. Before testing the first hypothesis, we examined whether the VHF tasks produced the expected visual field asymmetries in the control group using one-tailed t-tests that reflected the expected direction of the difference between visual fields. The tests confirmed the expected significant RVF advantage for words in the control group, for both accuracy, *t*(44) = 3.92, *p* < 0.001, *d* = 0.72, 95% CI [3.94, + ∞], and reaction time, *t*(44) = −2.28, *p* = 0.027, *d* = −0.11, 95% CI [-∞, −4.09]. The expected LVF advantage for the symmetry decision task was significant in reaction times for the control group, *t*(44) = −3.03, *p* = 0.004, *d* = −0.12, 95% CI [-∞, −7.88], however no significant advantage was found for accuracy, *t*(44) = 1.56, *p* = 0.127, *d* = 0.25, 95% CI [−0.21, + ∞].

**Table 3:** Difference scores (and SD) on the VHF tasks per group

Measure	Task	Group	Difference VFs	RBA
*Accuracy (%)*	LDT	Control	6.89 (11.78)	4.19 (4.85)
		Dyslexia	7.44 (13.33)	6.44 (5.42)
	SDT	Control	2.69 (11.61)	8.17 (7.24)
		Dyslexia	4.28 (9.73)	5.86 (6.39)
*RT (ms)*	LDT	Control	16.52 (45.62)	27.03 (27.46)
		Dyslexia	48.34 (117.30)	22.07 (58.58)
	SDT	Control	17.68 (39.13)	17.12 (26.61)
		Dyslexia	20.47 (57.85)	23.09 (46.22)

*SD* standard deviation, *VHF* visual half-field, *LVF* left visual field, *RVF* right visual field, *BVF* bilateral visual field, *RBA* redundant bilateral advantage, *RT* reaction time, *LDT* lexical decision task, *SDT* symmetry decision task, difference between visual fields was calculated as RVF-LVF for the LDT, and as LVF-RVF for the SDT, and all difference scores are summarized as absolute values

To test the first hypothesis, we calculated difference scores to examine whether the difference in performance between the visual fields was greater in the dyslexia group. There were no significant group differences in RVF-LVF differences when comparing the dyslexia and control group in the lexical decision task for accuracy, *t*(86.68) = 0.21, *p* = 0.835, *d* = 0.04, 95% CI [−3.85, + ∞], nor reaction times, *t*(57.02) = −1.75, *p* = 0.086, *d* = −0.37, 95% CI [-∞, −1.45]. We also found no significant group difference in the LVF-RVF differences for the symmetry decision task in accuracy, *t*(85.39) = 0.70, *p* = 0.485, *d* = 0.15, 95% CI [−2.17, + ∞], or reaction times, *t*(77.3) = −0.27, *p* = 0.790, *d* = −0.06, 95% CI [-∞, 14.55].

As our results on visual word processing differed from those of previous studies [7, 30], unplanned post hoc analyses were conducted. Bradshaw et al. [7] and Henderson et al. [30] found that the observed RVF advantage in their dyslexia samples could be explained by a reduced word accuracy in the LVF, but not a difference in the RVF, as compared to controls. Therefore, we used independent t-tests to compare accuracy and the reaction times to the LVF and the RVF between the groups for both tasks. Because these analyses were exploratory and not specified a priori, two-sided tests were used to allow for the possibility of differences in either direction. For the lexical decision task, accuracy was significantly lower in the dyslexia group compared to the control group for both the LVF, *t*(87.88) = −2.82, *p* = 0.024, *d* = −0.60, 95% CI [−11.60, −2.01], and the RVF, *t*(85.62) = −4.40, *p* < 0.001, *d* = −0.93, 95% CI [−9.07, −3.43]. In addition, reaction times were significantly longer in the dyslexia group compared to the control group in the LVF, *t*(71.89) = 3.90, *p* < 0.001, *d* = 0.82, 95% CI [74.44, 230.15], and the RVF, *t*(86.86) = 3.77, *p* = 0.001, *d* = 0.80, 95% CI [56.57, 182.39]. For the symmetry decision task, reaction times to the RVF trials were significantly longer in the dyslexia group, *t*(87.73) = 2.63, *p* = 0.040, *d* = 0.55, 95% CI [20.54, 147.76]. Reaction times to the LVF approached significance with longer reaction times in the dyslexia group, *t*(87.59) = 2.46, *p* = 0.063, *d* = 0.52, 95% CI [15.64, 147.09]. Accuracy did not significantly differ between groups for both the RVF, *t*(87.81) = 0.27, *p* = 1, *d* = 0.06, 95% CI [−3.87, 5.09], and the LVF, *t*(82.43) = 1.06, *p* = 1, *d* = 0.22, 95% CI [−1.91, 6.30].

#### Redundant bilateral advantage

**Fig. 3:**
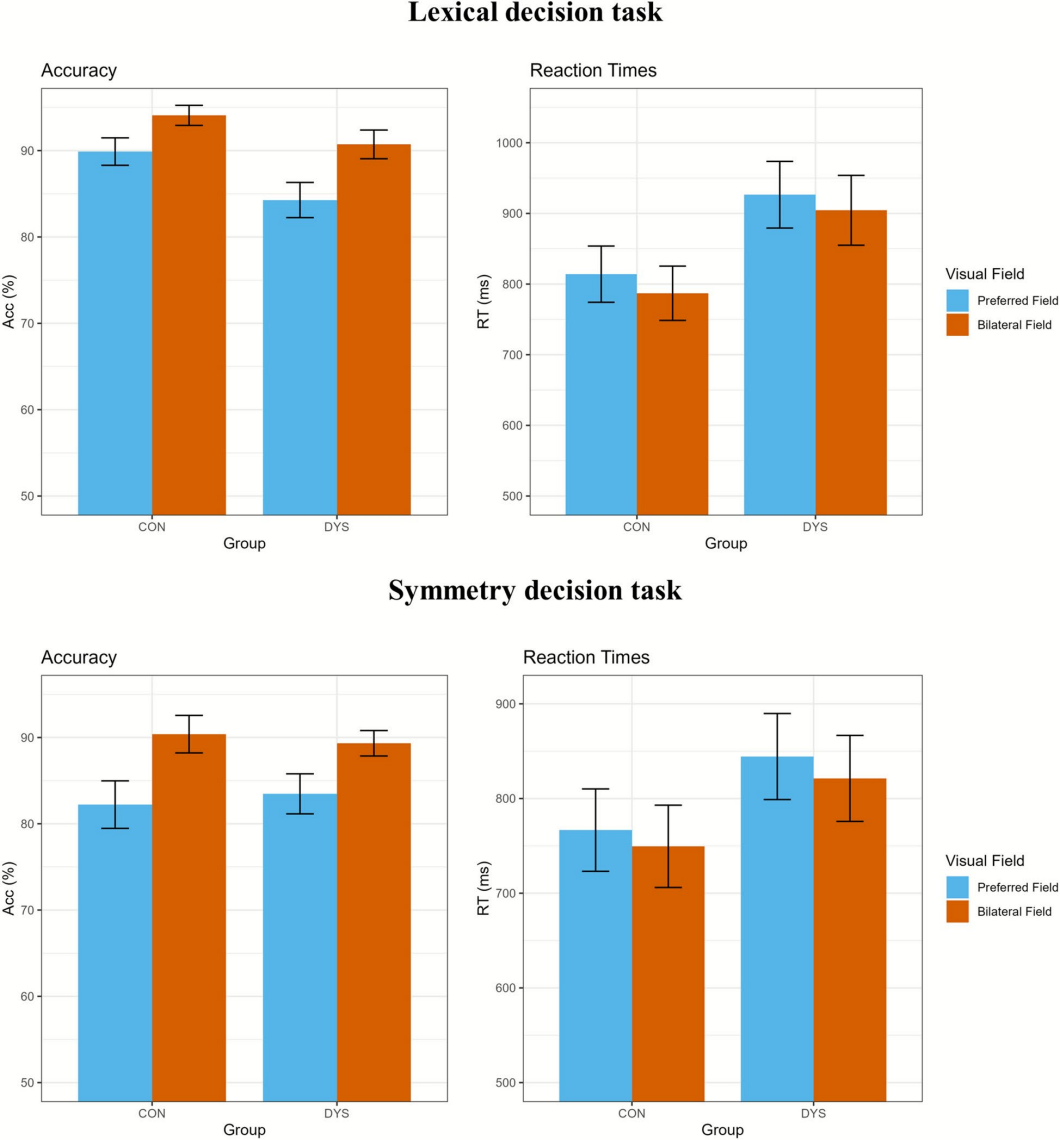
Bar plots of preferred and bilateral visual field of both tasks per group. Note: Bar plots of the mean accuracy (in %) and mean reaction times (in ms) for the preferred visual field and the bilateral visual field per group, 95% confidence intervals in black. Bars start at 50% accuracy and 500 ms reaction time. ACC = accuracy, RT = reaction time, CON = control group, DYS = dyslexia group

RBA scores are summarized for each group and task in Table 3, and mean accuracy and reaction times for the bilateral and preferred visual fields are displayed in Fig. 3. As shown in Supplementary Figures S3 and S4, the RBA varied within the dyslexia group, with some individuals showing larger differences. Before testing the second hypothesis, we verified whether the RBA was present in the control group using one-tailed t-tests that reflected the expected direction of the difference between conditions. A significant RBA, as compared to the preferred unilateral visual field, was confirmed in the control group for both the lexical decision task (accuracy: *t*(44) = 5.80, *p* < 0.001, *d* = 0.89, 95% CI [2.98, + ∞], reaction times: *t*(44) = −6.60, *p* < 0.001, *d* = −0.21, 95% CI [-∞, −20.15]) and the symmetry decision task (accuracy: *t*(44) = 7.56, *p* < 0.001, *d* = 0.97, 95% CI [6.35, + ∞], reaction time: *t*(44) = −4.32, *p* < 0.001, *d* = −0.12, 95% CI [-∞, −10.45]).

Based on the second hypothesis, it was expected that the RBA would be reduced in the dyslexia group compared to the control group. However, the RBA in the lexical decision task was larger in the dyslexia group based on accuracy (see Table 3), which was in the unexpected direction. The RBA, based on reaction times, was not significantly reduced in the dyslexia group, *t*(62.45) = 0.51, *p* = 0.601, *d* = 0.11, 95% CI [−11.14, + ∞]. The RBA was not significantly reduced in the symmetry decision task based on accuracy scores, *t*(86.65) = −1.60, *p* = 0.113, *d* = −0.34, 95% CI [-∞, 0.09], and the difference in RBA based on reaction times was to the unpredicted direction (larger in the dyslexia group; see Table 3).

**Fig. 4:**
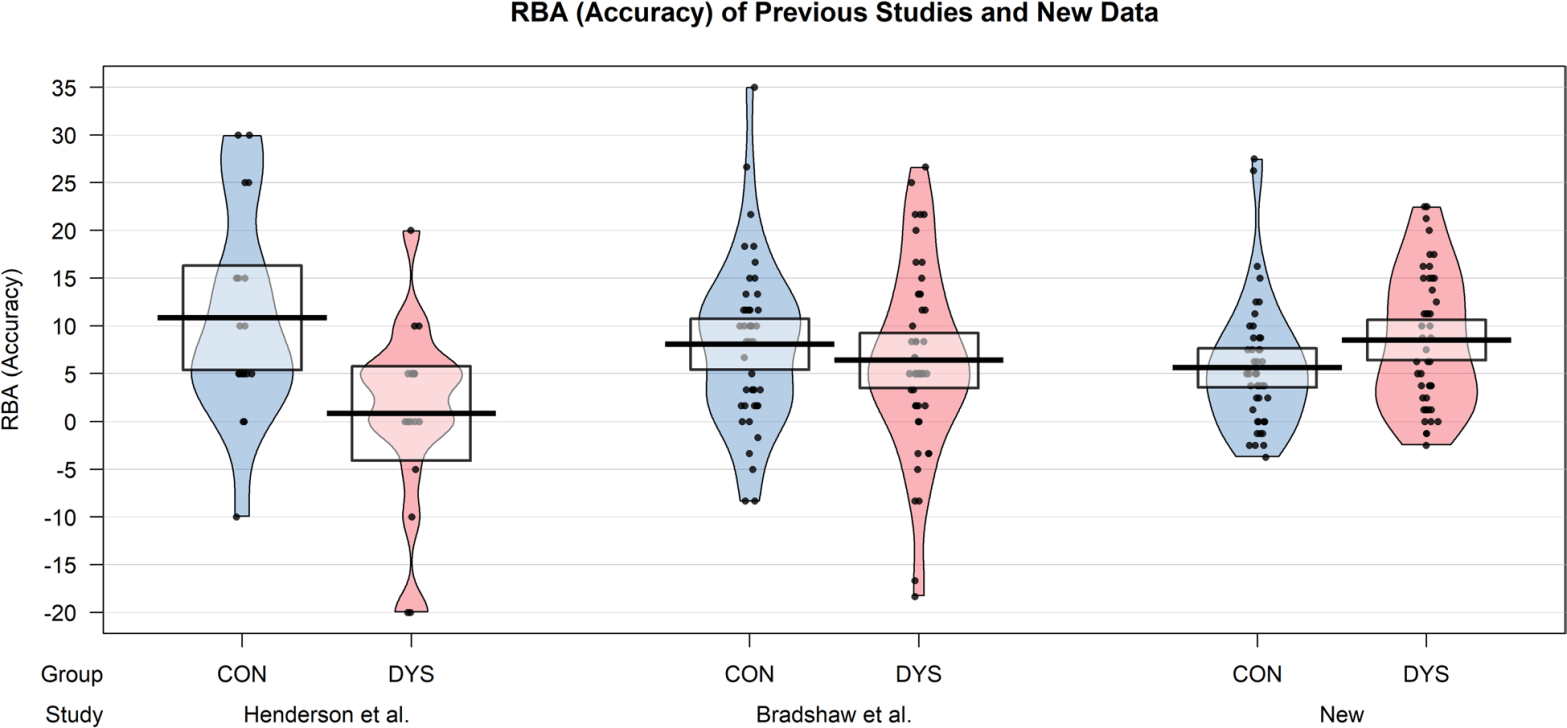
Redundant bilateral advantage (accuracy) of previous studies compared to new data of the lexical decision task. Note*:* Comparison of the RBA based on accuracy of the previous studies of Henderson et al. [30] and Bradshaw et al. [7], and the new data of the lexical decision task of the current study, visualized in pirate plots displaying the mean and individual data points of the two groups. The shaded boxes represent the 95% confidence intervals. Note that the visualization of the data of Henderson et al. [30] is based on the data of 18 participants per group, as not all data was available. RBA = redundant bilateral advantage, CON = control group, DYS = dyslexia group

As we calculated the RBA using each participant’s preferred visual field, whereas Henderson et al. [30] and Bradshaw et al. [7] used the RVF as a reference for the RBA for all participants, we conducted a post hoc exploratory analysis to ensure this did not account for the difference in results for the lexical decision task. In this analysis, the RBA was calculated using each participant’s RVF, consistent with Henderson et al. [30] and Bradshaw et al. [7]. This did not change the outcome of the tests, with the group difference for the RBA in the lexical decision task still being non-significant (accuracy: *t*(87.78) = 2.00, *p* = 1.000, *d* = 0.42, 95% CI [-∞, 5.30], reaction times: *t*(66.03) = −0.18, *p* = 1.000, *d* = −0.04, 95% CI [−21.19, + ∞]). A visual comparison of the data from Henderson et al. [30] and Bradshaw et al. [7], and our new data using the equivalent calculation method of the RBA, is presented in Fig. 4.

### Correlations with reading and spelling abilities

To examine the third hypothesis, partial correlation analyses were conducted to investigate the relationship between visual field differences in both visual half-field tasks and performance on independent reading and spelling tests in the dyslexia group, while controlling for sex and years of education. Correlations were calculated for the RVF-LVF difference in the lexical decision task and the LVF-RVF difference in the symmetry decision task, using both accuracy and reaction time. After Bonferroni correction, difference scores showed no significant correlation with any language test scores. Tables 4 and 5 summarize the correlations, and Figures S1 and S2 in the supplemental materials provide visualizations of the results.

**Table 4:** Pearson’s partial correlations (ρ) between RVF-LVF difference scores and RBA scores of the lexical decision task, and reading and spelling tests in the dyslexia group

	RVF-LVF Difference		RBA	
	Accuracy	RT	Accuracy	RT
spelling GL&SCHR (*p*)	.10 (1.000)	-.06 (1.000)	-.35 (.138)	-.002 (1.000)
phonological awareness (*p*)	-.22 (.936)	-.06 (1.000)	.36 (.108)	.20 (1.000)
LEMs reading test (*p*)	-.03 (1.000)	.12 (1.000)	-.02 (1.000)	-.13 (1.000)

*P*-values are Bonferroni corrected per asymmetry measure (RVF-LVF difference and RBA)

**Table 5:** Pearson’s partial correlations (ρ) between LVF-RVF difference scores and RBA scores of the symmetry decision task, and reading and spelling tests in the dyslexia group

	LVF-RVF Difference		RBA	
	Accuracy	RT	Accuracy	RT
spelling GL&SCHR (*p*)	-.14 (1.000)	-.14 (1.000)	-.32 (.312)	.02 (1.000)
phonological awareness (*p*)	.34 (.204)	-.20 (1.000)	.27 (.660)	.16 (1.000)
LEMs reading test (*p*)	-.09 (1.000)	.26 (.756)	-.26 (.720)	.03 (1.000)

*P*-values are Bonferroni corrected per asymmetry measure (RVF-LVF difference and RBA)

Furthermore, partial correlations were conducted between the RBA scores of both visual half-field tasks and the reading and spelling test scores in the dyslexia group, while controlling for sex and years of education. Correlations were calculated for the RBA in the lexical decision and symmetry decision tasks, using both accuracy and reaction times. After Bonferroni correction, RBA scores showed no significant correlation with language abilities. Tables 4 and 5 summarize the correlations, and scatterplots in Figures S3 and S4 in the supplemental materials visualize the results.

## Discussion

The current study examined the interhemispheric transfer deficit theory in dyslexia, which posits that dyslexia is associated with impaired hemispheric transfer, using two visual half-field paradigms. A lexical decision task measured both accuracy and reaction times of written word recognition, while a non-verbal symmetry decision task assessed whether interhemispheric transfer deficits extended beyond language, given that visuospatial abilities have been found to be impaired in dyslexia [13]. For both tasks, we tested three hypotheses. The first hypothesis predicted that the dyslexia group would show a greater RVF–LVF difference in accuracy and reaction times. The second hypothesis predicted that RBA of the dyslexia group in accuracy and reaction times would be reduced. Finally, the third hypothesis examined whether the magnitude of the RVF–LVF difference and the RBA were associated with the language test scores in the dyslexia group through partial correlation analyses. Our results did not provide support for any of the three tested hypotheses.

### Mixed evidence for interhemispheric transfer deficits across tasks

The lexical decision task successfully showed a RVF advantage in both accuracy and reaction times in the control group, confirming its sensitivity to RVF–LVF differences in written word processing. Similarly, control participants had a LVF advantage in both accuracy and reaction times for the symmetry decision task, confirming the task’s sensitivity to right hemisphere dominance in visual symmetry processing. However, the visual half-field differences in either task were not significantly larger in the dyslexia group than in the control group, failing to support the first hypothesis.

Despite the absence of significant group differences in VHF effects, the dyslexia group performed worse on the lexical decision task than the control group, consistent with previous studies that used a typing task to measure word processing [7, 30]. However, this decreased performance could not be attributed only to impaired LVF processing, as both LVF and RVF trials were unexpectedly affected to the same extent. These results contrast with those of Henderson et al. [30] and Bradshaw et al. [7], in which LVF performance was impaired in the dyslexia group, whereas RVF performance remained comparable to that of controls. A notable difference across these studies is the tasks used: while Henderson et al. [30] and Bradshaw et al. [7] both used a typing word reproduction task, we used a lexical decision task. It is possible that the interhemispheric transfer deficit in dyslexia is task-specific, with different tasks tapping into distinct cognitive processes. For example, a lexical decision task requires the evaluation of words and non-words based on lexical information like orthography, phonology, and semantics [3, 50, 53], while a typing task requires the orthographic reproduction of presented words [17]. Thus, the typing task emphasizes word (re)production, while word production is not necessarily required in the lexical decision task. It is possible that previous studies found evidence for the interhemispheric transfer deficit theory in dyslexia because it applies specifically to word (re)production, but not to other lexical processes involved in a lexical decision task.

The dyslexia group did not show lower accuracy on the symmetry decision task but did show longer reaction times that approached significance. Again, we found no evidence of impaired transfer in the dyslexia group for this task. These results are inconsistent with those of Daini et al. [18] and Facoetti et al. [22], who reported impaired transfer in children with dyslexia across different visuospatial tasks. However, they align with the findings of Velay et al. [63], who, like this study, found no evidence of impaired transfer in a visuomanual pointing task. Studies on visuo-spatial processing have also employed a diverse range of tasks, each possibly engaging distinct cognitive and perceptual mechanisms. For the symmetry decision task used in this study, the participants only had to assess symmetry along the vertical axis, whereas Daini et al. [18] used a same-different orientation judgment task where participants had to assess the orientation of two identical images across either the vertical or the horizontal axis. The latter task is thus more extensive and might place different demands on attentional control, working memory, and perceptual integration. Facoetti et al. [22] used tasks on visual spatial attention, which in turn could involve different visuo-spatial processes compared to symmetry or orientation detection. Velay et al. [63] also used a different task, namely a visuo-manual pointing tasks, which tap more into motor processes. Taken together, the variety in tasks used to assess interhemispheric transfer deficits in visuo-spatial processing could also explain the inconsistent results. Future research should identify the processing mechanisms implicated in dyslexia by using tasks targeting specific aspects of word processing, visuospatial, and other non-verbal processes. More refined experimental paradigms could clarify whether interhemispheric transfer deficits are limited to certain cognitive domains or extend across tasks.

Another noticeable difference between these studies is the participants’ age. While Daini et al. [18] and Facoetti et al. [22] focused on children, Velay et al. [63] and the current study used adult participants. There may be a developmental factor involved in the observation of an interhemispheric transfer deficit in visuospatial tasks, since studies involving children yielded positive results in favor of the theory, while studies focusing on adults did not. It is possible that adults adapted processing strategies for these tasks to overcome the deficit observed in children. It could also be that adults are better at performing these types of tasks than children, as can be inferred from the high accuracy rates in the current study, resulting in less noticeable observable differences in performance between the visual half-fields. However, in this case, it applies only to visuospatial tasks. The other studies on word-processing [7, 30] and the current study all involved adults and thus do not support the idea that a developmental factor could explain differences in results regarding interhemispheric transfer deficits in word-processing.

Taken together, the literature presents contradictory results, with some studies reporting evidence in favor of a deficit in hemispheric transfer in dyslexia, while others, like this study, do not. Another factor that could account for this is the use of small sample sizes in some studies, which may have led to an overestimation of the differences between the dyslexia group and the control group [12]. Studies with small sample sizes are more susceptible to sampling error and spurious results, potentially distorting the overall picture presented in the literature. As such, caution is needed when interpreting results from small sample size studies in isolation. Using larger and representative samples across tasks should give us a more accurate understanding of the conditions under which the interhemispheric transfer deficit might occur in dyslexia.

Although the planned analyses did not reveal group differences in visual half-field differences, the post-hoc group comparisons of performance to each field separately could provide additional context to interpret the results. The dyslexia group showed lower accuracy and longer reaction times than the control group to both words presented to the LVF and the RVF. This is in contrast with the findings of Bradshaw et al. [7] and Henderson et al. [30], who found a LVF specific deficit. Our results suggest that word recognition difficulties might be due to a more general deficit rather than a hemisphere-specific impairment. Exploratory analyses of the symmetry decision task showed longer reaction times in the dyslexia group, with a significant difference for RVF trials and a trend for LVF trials, while accuracy was not significantly different between groups. These findings do not align with a hemisphere-specific transfer deficit, but instead suggest a more general slowing of symmetry decision-making. These exploratory findings should be interpreted cautiously but highlight the importance of distinguishing between hemisphere-specific and global processing deficits in individuals with dyslexia in future research.

### Unexpected redundant bilateral advantage in dyslexia

Both tasks revealed a significant RBA in accuracy and reaction times in the control group, supporting their validity in measuring a RBA in both written-word and visual-symmetry processing. However, in both tasks, the dyslexia group’s RBA was not significantly smaller than that of the control group, failing to support the second hypothesis. These results are inconsistent with the findings of Henderson et al. [30], who observed an absence of the RBA in their dyslexia group. However, Bradshaw et al. [7] also found an RBA in their dyslexia group compared to controls, consistent with our findings. Based on visual inspection of our data, the RBA appears to be increased in the dyslexia group in some conditions, especially for accuracy in the lexical decision task (see Fig. 3). This finding is surprising, as the interhemispheric transfer hypothesis would predict an absent or reduced RBA [2, 41]. We did, however, not test if the effect was significant, as our predictions were in the opposite direction. These results cannot be explained by a lack of differences in language proficiency between the groups, as the dyslexia group performed significantly worse on validated dyslexia discrimination tests [61]. Instead, the current results may be explained by the unexpectedly poorer performance on words in the RVF in the dyslexia group. Since most participants preferred the RVF and this condition was also impaired in the dyslexia group, the difference between performance in the preferred field and performance with bilaterally presented words may have been exaggerated, leading to an apparent increase in the RBA in the dyslexia group. This could also explain why our findings were in the opposite direction to those of Bradshaw et al. [7] and Henderson et al. [30], in which performance on words in the RVF was not affected in the dyslexia group. Of course, a noticeable difference lies in the calculation of the RBA. Henderson et al. [30] and Bradshaw et al. [7] both used the RVF as a reference, even though some participants showed higher accuracy in the LVF. This approach risks overestimating the RBA, potentially inflating the advantage when individual differences in lateralization are not accounted for. To address this, the current study identified each participant’s preferred hemisphere/visual field and calculated the RBA as the difference between performance in the preferred and bilateral fields. While this method prevents overestimation, it does not explain the conflicting results, as recalculating the RBA using prior methods yielded consistent outcomes. Even when we adopted the same approach used by Henderson et al. [30] and Bradshaw et al. [7], the trend favoring the dyslexia group remained. In both our original analysis and the recalculation using their method, the dyslexia group showed a larger RBA for accuracy on the lexical decision task (see Fig. 4). This suggests that the unexpected pattern is not due to differences in how the RBA was calculated, but is genuinely reflected in the data. Further investigation is needed to replicate this unexpected finding and to assess in which context individuals with dyslexia experience an increased or reduced RBA.

### Correlation with language abilities

If impaired interhemispheric transfer is related to dyslexia symptoms, we would expect the size of the delay to be related to the severity of dyslexia symptoms. However, partial correlation analysis revealed no significant relations between the reading, spelling, and phonological awareness scores and the LVF-RVF difference or with the RBA of both tasks in the dyslexia group. It is important to note that ceiling effects were observed in the VHF task data and that the VHF method is an indirect behavioral method not considered sensitive enough to determine language lateralization on the individual level [31]. This limits the reliability of the correlation analysis. Given these limitations, the results of the correlation analyses should be interpreted with caution.

### Limitations of visual half-field tasks and future research

Although the VHF paradigm remains a valuable behavioral method for examining hemispheric lateralization and interhemispheric transfer, a few possible limitations of the present implementation should be noted. Firstly, the tasks appeared to be very simple, as indicated by the high accuracy scores. Accuracy ranged from 75 to 90% in the dyslexia group and from 75 to 95% in the control group. Ceiling effects can lead to underestimated variability [60], and as such, the differences between the control and dyslexia groups may also have been underestimated, since lower variability can reduce the likelihood of detecting group differences [57]. Future research could implement the same tasks with more challenging items to examine how this affects variance and the observed group differences.

Secondly, another limitation is the lack of control for eye movements away from the fixation cross. It cannot be guaranteed that stimuli were consistently presented in the intended visual half-field if participants had difficulty maintaining fixation. It would have been preferable to control for eye movements using eye-tracking. An interesting aspect for future research would be to test the relationship between stimulus presentation time, eye movements away from the fixation cross, and performance on a VHF task. Here, we opted for the upper limit of recommended stimulus presentation times [31]. Longer presentation times could have increased the likelihood of eye movements toward the relevant stimulus, whereas shorter times might have impaired performance due to reduced stimulus clarity.

It is also important to note that VHF tasks serve only as behavioral proxies for hemispheric asymmetry and can be unreliable at the individual level due to high variability in performance [31]. Functional brain imaging offers a more robust method for identifying individual lateralization patterns (e.g. [32, 34]), although VHF tasks remain reliable at the group level [47]. To more directly examine interhemispheric transfer times in individuals with dyslexia, future research could capitalize on EEG’s high temporal resolution, which allows for a more precise assessment of the timing of interhemispheric communication at the individual level [24]. Nevertheless, observing changes in behavior may be more functionally relevant than neurophysiological differences, particularly when the goal is to understand or improve cognitive performance [44]. Thus, we argue that the present findings support the use of VHF tasks as an appropriate behavioral tool for studying hemispheric transfer at the group level, and a low-cost alternative to neuroimaging techniques.

## Conclusions

The current study provides new insights into the interhemispheric transfer deficit theory in dyslexia. While both tasks effectively captured lateralization patterns in the control group, no evidence of significantly larger asymmetries or reduced redundant bilateral advantages was found in the dyslexia group. These findings challenge the interhemispheric transfer deficit theory in dyslexia across two different cognitive domains. The results emphasize the need for task-specific investigations, as observed interhemispheric transfer deficits may vary depending on the processes involved. Despite its limitations, this study underscores the importance of task design in advancing our understanding of dyslexia and lateralization. Clinically, these results suggest that behavioral visual half-field tasks may provide complementary insights into hemispheric processing, but have limited utility for the assessment of dyslexia, and should not replace standard phonological and reading assessments.

## Supplementary Information

Supplementary Material 1.

## Data Availability

The data that supports the findings of this study are openly available in OSF at https://osf.io/qgehx/.
