# Supplementary material for: Hemispheric transfer and dyslexia: testing the deficit hypothesis for word and symmetry recognition using visual half-field tasks
Source: J Neurodev Disord. 2026 Jan 29;18:10. doi: 10.1186/s11689-026-09674-4 (PMC12924301; doi:10.1186/s11689-026-09674-4)
Supplement: Supplementary file 1 — Supplementary Material 1. [file 11689_2026_9674_MOESM1_ESM.docx]

**Supplement to manuscript “Hemispheric transfer and dyslexia: Testing the deficit hypothesis for word and symmetry recognition using visual half-field tasks”**

| **Figure S1**  *Partial correlations between RVF-LVF difference scores of the lexical decision task, and reading and spelling tests in the dyslexia group* | |
| --- | --- |
| 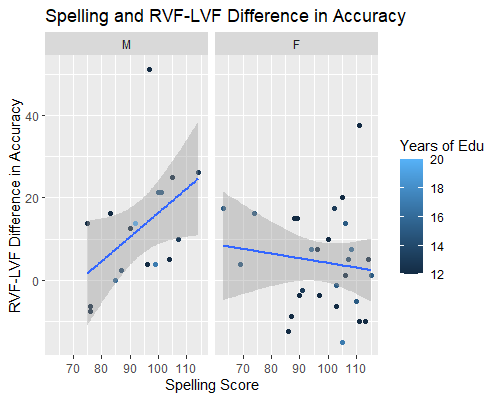 | 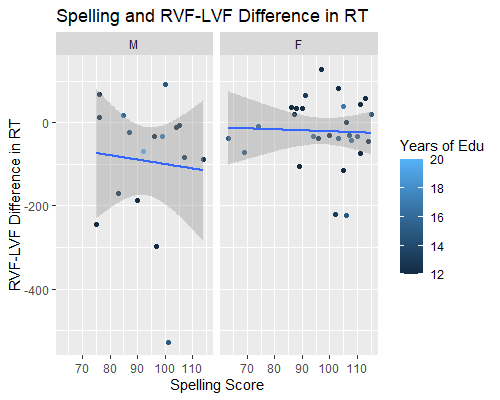 |
| 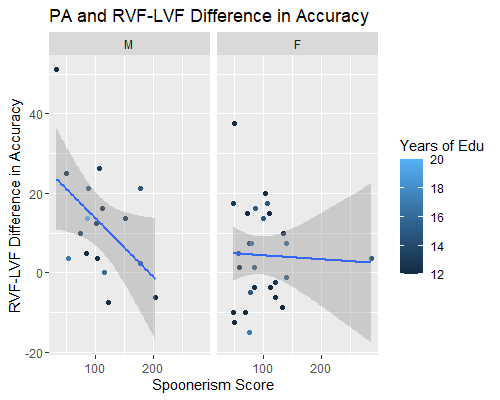 | 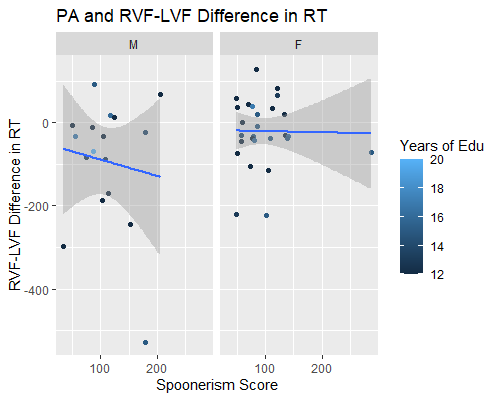 |
| 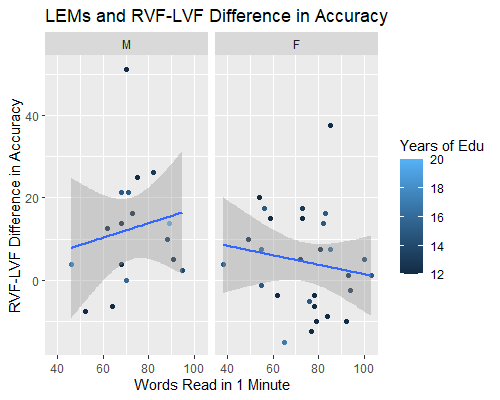 | 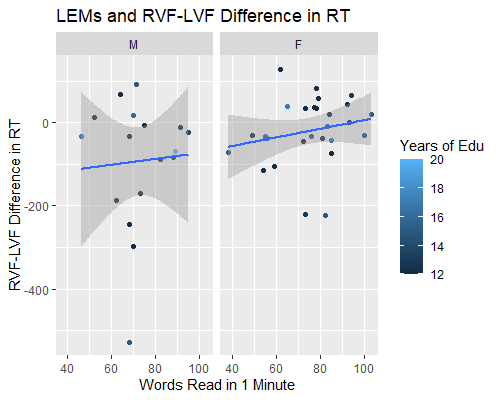 |
| *Note:* Scatterplots of the correlation between the RVF advantage based on accuracy and reaction times, and the independent reading and spelling tests, taking into account sex (M = male, F = female) and the number of years of education. The blue line represents the fitted regression line, and the grey area the 95% confidence interval around the fitted regression line. RVF = right visual field, Years of Edu = number of years of formal education, RT = reaction time, PA = phonological awareness, LEMs = Leestest 1-minuut studenten (Word Reading Test for students) | |
| \| **Figure S2**  *Partial correlations between LVF-RVF difference scores of the symmetry decision task, and reading and spelling tests in the dyslexia group* \| \| \| --- \| --- \| \| 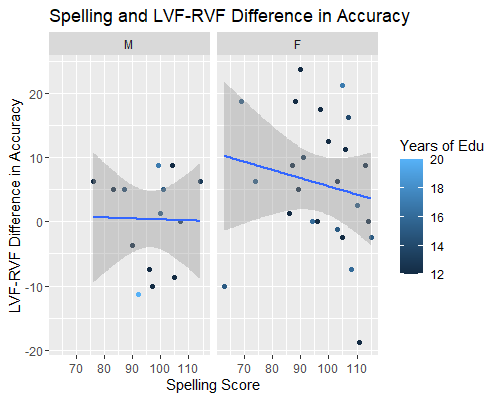 \| 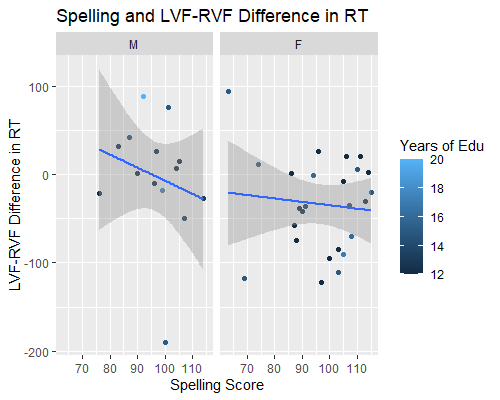 \| \| 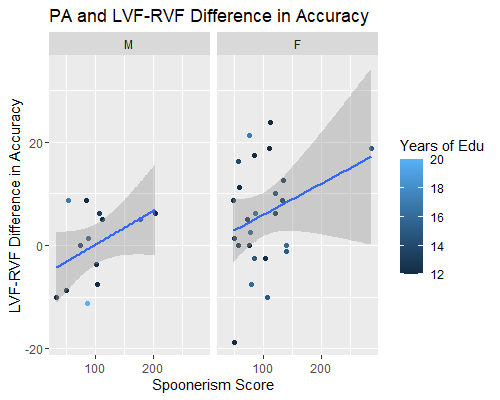 \| 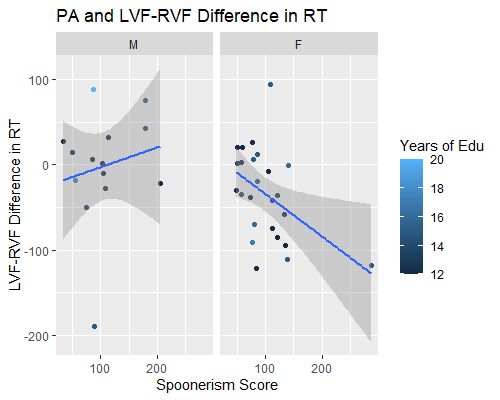 \| \| 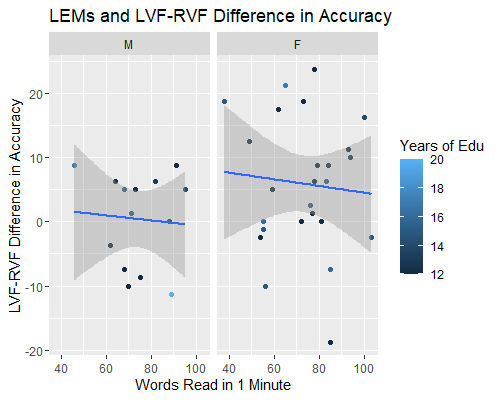 \| 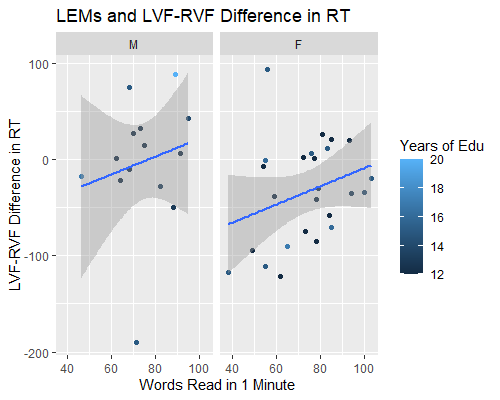 \| \| *Note:* Scatterplots of the correlation between the RVF advantage based on accuracy and reaction times, and the independent reading and spelling tests, taking into account sex (M = male, F = female) and the number of years of education. The blue line represents the fitted regression line, and the grey area the 95% confidence interval around the fitted regression line. RVF = right visual field, Years of Edu = number of years of formal education, RT = reaction time, PA = phonological awareness, LEMs = Leestest 1-minuut studenten (Word Reading Test for students) \| \| | |

| **Figure S3**  *Partial correlations between RBA scores of the lexical decision task, and reading and spelling tests in the dyslexia group* | |
| --- | --- |
| 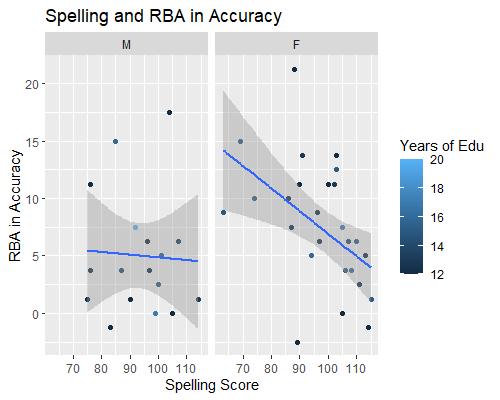 | 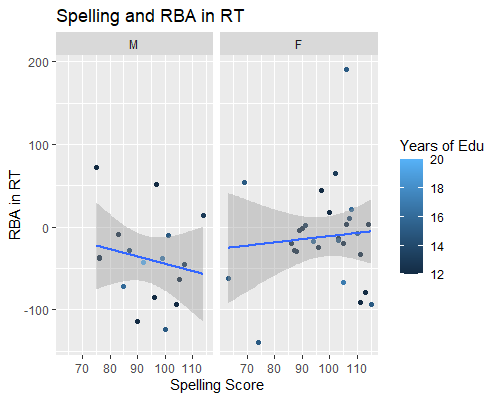 |
| 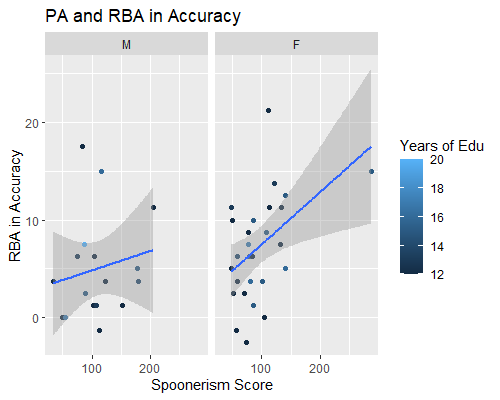 | 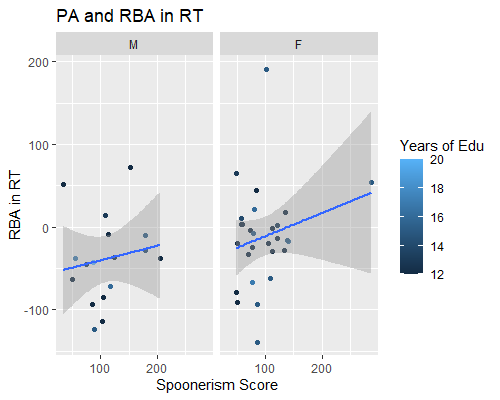 |
| 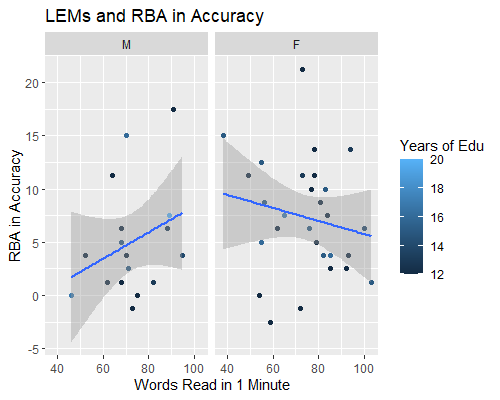 | 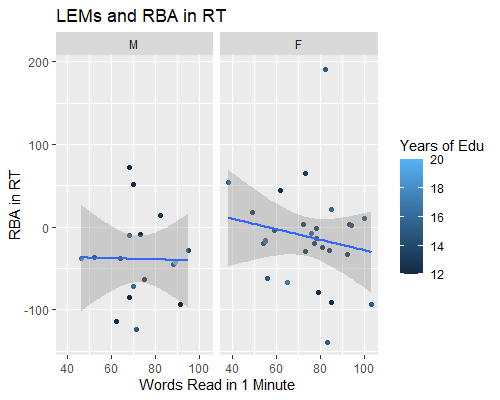 |
| *Note:* Scatterplots of the correlation between the RBA based on accuracy and reaction times, and the independent reading and spelling tests, taking into account sex (M = male, F = female) and the number of years of education. The blue line represents the fitted regression line, and the grey area the 95% confidence interval around the fitted regression line. RBA= redundant bilateral advantage, Years of Edu = number of years of formal education, RT = reaction time, PA = phonological awareness, LEMs = Leestest 1-minuut studenten (Word Reading Test for students) | |

| **Figure S4**  *Partial correlations between RBA scores of the symmetry decision task, and reading and spelling tests in the dyslexia group* | |
| --- | --- |
| 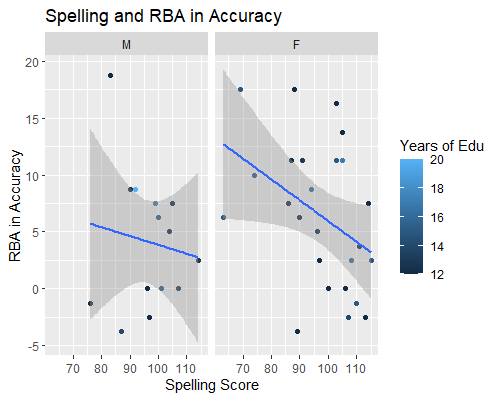 | 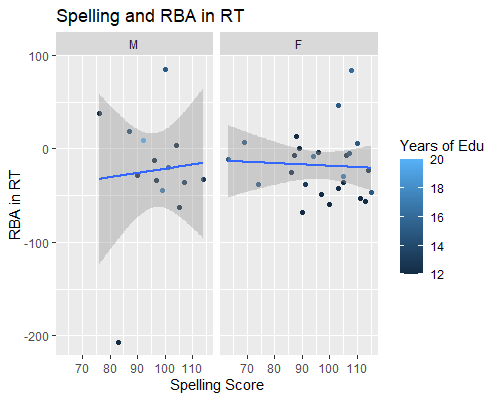 |
| 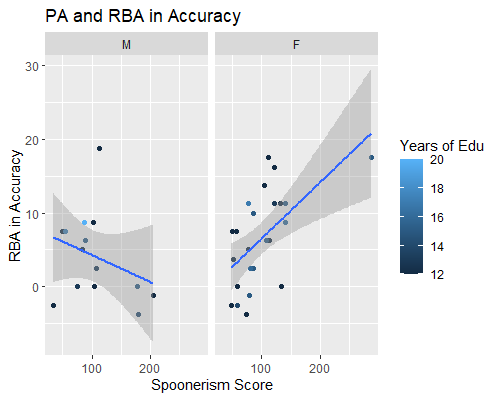 | 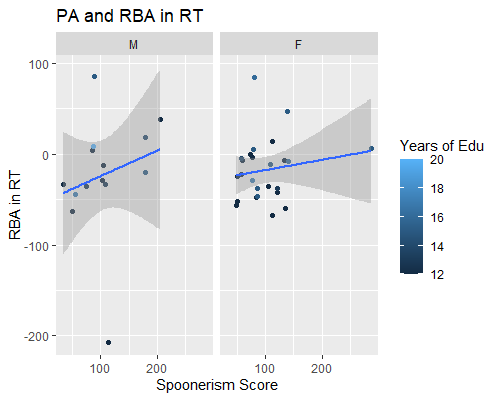 |
| 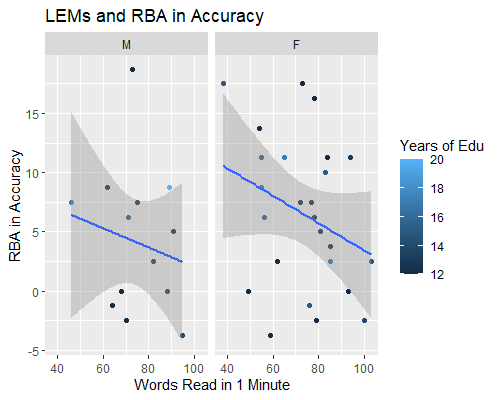 | 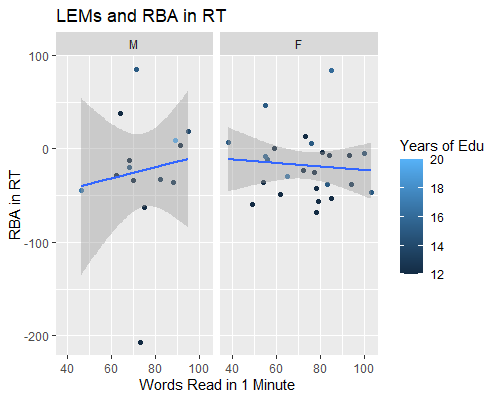 |
| *Note:* Scatterplots of the correlation between the RBA based on accuracy and reaction times, and the independent reading and spelling tests, taking into account sex (M = male, F = female) and the number of years of education. The blue line represents the fitted regression line, and the grey area the 95% confidence interval around the fitted regression line. RBA = redundant bilateral advantage, Years of Edu= number of years of formal education, RT = reaction time, PA= phonological awareness, LEMs = Leestest 1-minuut studenten (Word Reading Test for students) | |
